# The impact of the COVID-19 pandemic on hospital-acquired infections and multi-drug resistant organisms, in comparison to seasonal influenza

**DOI:** 10.1186/s12879-024-09240-0

**Published:** 2024-11-28

**Authors:** Halima Dabaja-Younis, Zmira Silman, Jalal Tarabeia, Khetam Hussein

**Affiliations:** 1 Paediatric Infectious Diseases Unit, Rambam Health Care Campus, Haifa, Israel; 2 Infection Prevention and Control Unit, Rambam Health Care Campus, P.O. Box 9602, Haifa, 31096 Israel; 3 The Ruth & Bruce Rappaport Faculty of Medicine, Technion–Israel Institute of Technology, Haifa, Israel; 4 Independent statistics consultant, Netanya, Israel; 5 The Max Stern Yezreel Valley College, Affola, Israel

**Keywords:** Multi-drug, Resistant microorganisms, *Clostridioides difficile*, COVID-19, Influenza

## Abstract

**Background:**

While effective preventive measures reduce hospital-acquired infections (HAIs) and the spread of multi-drug resistant organisms (MDROs), studies on the impact of the COVID-19 pandemic and its associated preventive measures remain inconclusive.

**Objective:**

To assess the impact of COVID-19 on HAIs and MDROs and to compare it with the effect of seasonal influenza.

**Methods:**

A retrospective cohort study analyzed prospectively collected data from a tertiary hospital in Haifa, northern Israel, from 2016 to 2021. It compared pre/during COVID-19 and influenza (Dec-Feb)/non-influenza (Mar-Nov) seasons. Studied parameters: hospital-acquired bloodstream infections (HA-BSI), MDROs, nosocomial *Clostridioides difficile* infections (CDI) per 10,000 hospital days (HD), central line-associated BSI (CLABSI) per 1000 catheter days (CD) and hand hygiene compliance (HHC).

**Results:**

During the COVID-19 period, rates of HAIs and MDROs decreased compared to the pre-COVID era for methicillin-resistant *Staphylococcus aureus* (MRSA) (4.2 vs. 6.9/10,000 HD; *p* < 0.001), carbapenem-resistant *Acinetobacter baumani* (CRAB) (2.2 vs. 3.1/10,000 HD; *p* = 0.02), and nosocomial CDI (3 vs. 4.6/10,000 HD; *p* < 0.001). However, there was a higher rate of carbapenem-resistant Enterobacteriaceae (CRE) (4.6 vs. 2.7/10,000 HD; *p* < 0.001) and HA-BSI (29.7 vs. 27.3/10,000 HD; *p* = 0.006) during the COVID-19 era. CLABSI rates showed no significant difference (2.3 vs. 2.7/1000 CD; *p* = 0.910). HHC rate remained at 70% in both eras (*p* = 0.151). No significant differences were observed in MDROs, CDI, HA-BSI, or CLABSI rates (*p* = 0.233, 0.675, 0.267, and 0.563, respectively) between influenza and non-influenza seasons.

**Conclusions:**

In the COVID-19 era, HAIs and MDROs rates significantly declined, while CRE rates increased, possibly due to a national trend in Israel since 2016. Steady HHC rates suggest additional factors like enhanced environmental cleaning, personal protective equipment usage, and increased infection prevention awareness contributed to this decline. Influenza had no noticeable impact, likely due to healthcare workers’ varying perceptions and the brevity of the influenza season, making it challenging to assess impact.

## Background

Since the emergence of coronavirus disease 2019 (COVID-19) in December 2019, healthcare institutions have adjusted their standard precautions to address the ongoing respiratory morbidity challenges. Hospitals have implemented adjusted infection prevention and control (IPAC) measures, this includes widespread mask usage, isolating COVID-19 patients in dedicated units or private rooms, using personal protective equipment during treatment, enhancing awareness of hand hygiene compliance (HHC), and intensifying environmental cleaning practices [1, 2].

Enhancing public and healthcare system awareness of IPAC measures in response to COVID-19 is expected to yield favorable results in reducing hospital-acquired infections (HAIs) and the acquisition of multidrug-resistant organisms (MDROs), as demonstrated in several studies [3–5]. Nevertheless, the ongoing challenge of MDROs cross-transmission and HAIs remains a substantial burden for healthcare facilities, as indicated by other research findings [6–8].

Several factors have been identified as contributing to this paradoxical impact of COVID-19 on HAIs and MDROs rates. The surge in COVID-19 patients, coupled with an ongoing shortage of healthcare workers (HCWs) due to isolation following COVID-19 infection or contact with confirmed cases [9] led to the recruitment of less experienced HCWs. Additionally, the general exhaustion and burnout experienced by HCWs, along with the COVID-19 psychological and physical toll, exacerbated by the challenging working conditions [10–12], have played a role in this situation.

This study aimed to assess whether the COVID-19 pandemic had an impact on the rates of MDROs, hospital-acquired bloodstream infections (HA-BSI), nosocomial *Clostridioides difficile* infections (CDI), and hand hygiene compliance (HHC) rates among healthcare workers (HCWs). This impact was compared to that of seasonal influenza.

## Methods

Design: A retrospective study of prospectively collected data.

Setting: The study was conducted at Rambam Health Care Campus (RHCC), a 1000-bed tertiary university-affiliated hospital serving a catchment area of more than two million citizens in northern Israel. During the pandemic, up to six departments, including the intensive care unit, were dedicated to the admission of confirmed COVID-19 patients. Except for one, these departments were closed to admissions between COVID-19 peak periods.

Participants: All patients, both adults and children, who were admitted to RHCC from January 2016 to December 2021, contributed their hospital days and catheter days for evaluating MDROs and HAIs incidence.

### Measured outcomes

1. The rate of hospital-acquired MDROs, CDI, and BSI, including central line-associated BSI (CLABSI) events. These rates were expressed as the number of patients who newly acquired these infections per 10,000 hospital days (HD). CLABSI events were calculated per 1,000 catheter days (CD).

MDROs encompassed methicillin-resistant *Staphylococcus aureus* (MRSA), carbapenem-resistant Enterobacterales (CRE), carbapenem-resistant *Acinetobacter baumannii* (CRAB), carbapenem-resistant *Pseudomonas aeruginosa* (CR-PA), and vancomycin-resistant Enterococci (VRE), which were identified more than 48 h after admission. Acquisition rates for MDROs included both colonization and clinical samples. Hospital-acquired bloodstream infections (HA-BSI) encompassed all bloodstream infections that occurred 48 h or later after admission. Central line-associated bloodstream infection (CLABSI) was defined as a bloodstream infection that occurred when the central catheter was used within 48 h before the onset of infection, and no other secondary focus was identified. CDI was categorized as hospital-acquired if positive specimens were collected 72 h or more after admission, or within 4 weeks from discharge in readmitted patients who had not been hospitalized in another healthcare facility during that time.

2. Hand hygiene compliance was measured by direct audits according to the hand hygiene technical reference manual published by the World Health Organization (WHO) [13].

HHC was assessed through direct observations conducted by trained observers with diverse backgrounds. These audits occurred in all hospital departments, covering both weekdays and weekends, as well as all work shifts. Each hand hygiene opportunity was documented in a standardized form.

The study period was divided into two distinct periods: the pre-COVID-19 pandemic era, spanning from January 1, 2016, to February 29, 2020, and the COVID-19 pandemic era, spanning from March 1, 2020 to December 31, 2021. Furthermore, the aforementioned metrics were compared between influenza seasons (winter season, occurring from December to February) and non-influenza seasons (non-winter season, occurring from March to November) within the study period.

Notably, due to the absence of influenza morbidity during the winter of 2020–2021, the period from December to March 2020–2021 was categorized as part of the non-influenza season for analysis purposes.

### Data sources

Data were obtained from the IPAC unit monthly surveillance databases. IPAC team conducts regular surveillance of MDROs, HA-BSI, and HHC audits.

### Microbiological methods

MRSA, VRE, CRAB, and CR-PA were determined according to the Vitek-2 automated system should be in continuity with the previous raw VITEK-2 without any interruptions and according to the CLSI breakpoints [14]. Samples of Clinical and surveillance MDROs cultures were processed according to national Israeli guidelines (2013) and according to CLSI criteria [15]. Toxin-producing *C. difficile* was determined based on a GDH-based serology test (C. DIFF QUIK CHEK COMPLETE^®^; Alere™) and in inconclusive testing were confirmed by a PCR-based test (Xpert^®^ C. difficile; Cepheid^©^). 

### Statistical analysis

Continuous variables were expressed as median and interquartile range (IQR). Categorical variables were expressed as numbers and percentages (%). Comparisons between periods, influenza/non-influenza season and pre/during COVID-19 were performed using Mann-Whitney U for continuous variables and Fisher’s exact test or Chi^2^ test for categorical variables.

Graphical representation is based on boxplots, depicting groups of numerical data through their quartiles and whiskers indicating variability outside the upper and lower quartiles. A two-sided *p-value* less than 0.05 was considered to define statistical significance. Analyses were carried out using in R-3.6.3 (R Foundation for Statistical Computing, Vienna, Austria).

## Results

The period preceding COVID-19 (Jan 2016 - Feb 2020) included 50 months, while the COVID-19 period (Mar 2020 - Dec 2021) included 22 months. The median hand hygiene compliance (HHC) audits were 573.5 (343-1125.8) pre-COVID-19 and 774.5 (616.2–1119) during COVID-19, resulting in a *p*-value of 0.165. Throughout both periods, the HHC rate remained stable at 70%, and noncompliance with glove use consistently stood at 40% (Table 1).

**Table 1:** The differences in median acquisition rates of multi-drug resistant organisms, hand hygiene compliance, and hospital-acquired bloodstream infections before and during the COVID-19 pandemic

	Pre COVID-19 pandemic (Jan 2016 - Feb 2020)	During COVID-19 pandemic (Mar 2020 - Dec 2021)	Total	*p*-value
Number of hospital days	1,338,206	555,252	1,893,458	
HHC rate [median (Q1-Q3)]	0.7 (0.6–0.8)	0.7 (0.7–0.8)	0.7 (0.6–0.8)	0.151
Non-compliance due to gloves donning [median (Q1-Q3)]	0.4 (0.3–0.5)	0.4 (0.4–0.5)	0.4 (0.3–0.5)	0.4485
HHC opportunities [median (Q1-Q3)]	573.5 (343-1125.8)	774.5 (616.2–1119)	654.0 (400.8-1134.2)	0.165
Overall HHC opportunities	39,906	18,751	58,657	
CRE/10,000 HD [median (Q1-Q3)]	2.7 (2.0-3.7)	4.6 (3.2–6.1)	3.1 (2.3–4.7)	**< 0.001**
MRSA/10,000 HD [median (Q1-Q3)]	6.9 (6.0-8.3)	4.2 (3.4–5.1)	6.3 (4.8–7.5)	**< 0.001**
CRAB/10,000 HD [median (Q1-Q3)]	3.1 (1.9–4.8)	2.2 (1.3–2.8)	2.5 (1.7–4.3)	**0.020**
CR-PA/10,000 HD [median (Q1-Q3)]	4.3 (3.4–4.9)	4.5 (3.9–4.9)	4.3 (3.6-5.0)	0.478
VRE/10,000 HD [median (Q1-Q3)]	0.4 (0.0-0.7)	0.4 (0.0-0.7)	0.4 (0.0-0.7)	0.704
CDI/10,000 HD [median (Q1-Q3)]	4.6 (3.1–5.7)	3.0 (1.8–4.1)	3.9 (2.9-5.0)	**< 0.001**
All MDROs [median (Q1-Q3)]	17.5 (15.9–20.3)	16.0 (14.0-17.9)	17.1 (14.9–20.0)	**0.049**
All MDROs & CDI [median (Q1-Q3)]	22.9 (20.0-25.6)	18.8 (16.8–21.8)	21.5 (18.6–24.7)	**0.001**
HA-BSI/10,000 HD [median (Q1-Q3)]	27.3 (23.7–30.3)	29.7 (27.9–33.1)	27.8 (24.1–31.2)	**0.006**
CLABSI/1,000 CD [median (Q1-Q3)]	2.7 (1.5–4.3)	2.3 (1.5–4.5)	2.7 (1.5–4.4)	0.910
Overall catheter days	41,583	17,752	59,335	

HHC- hand hygiene compliance, CRE- carbapenem-resistant *Enterobacterales*, MRSA*-* methicillin-resistant *Staphylococcus aureus*, CRAB- carbapenem-resistant *Acinetobacter baumani*, CR-PA-Carbapenem-resistant *Pseudomonas aeruginosa*, VRE- vancomycin-resistant *Enterococcus*. CDI- *Clostridioides difficile* infections

During the COVID-19 pandemic, MRSA and CRAB rates decreased significantly compared to pre-pandemic levels. MRSA went from 6.9 to 4.2 per 1000 HD (*p* < 0.001), and CRAB rate decreased from 3.1 to 2.2 per 1000 HD (*p* = 0.02). CDI rates also dropped from 4.6 to 3 per 10,000 HD (*p* < 0.001).

Overall, all MDRO and CDI rates were lower during COVID-19 (18.8 per 10,000 HD) compared to pre-COVID-19 pandemic (22.9 per 10,000 HD, *p* = 0.001). In contrast, CRE rates increased significantly during COVID-19 (4.6 per 10,000 HD) compared to the pre-pandemic period (2.7 per 10,000 HD, *p* < 0.001).

The HA-BSI rate was higher during the COVID-19 period (29.7 per 10,000 HD) compared to the pre-pandemic period (27.3 per 10,000 HD) with a significant difference (*p* = 0.006). However, CLABSI rates remained unchanged during both study periods (*p* = 0.910). (Table 1).

**Fig. 1:**
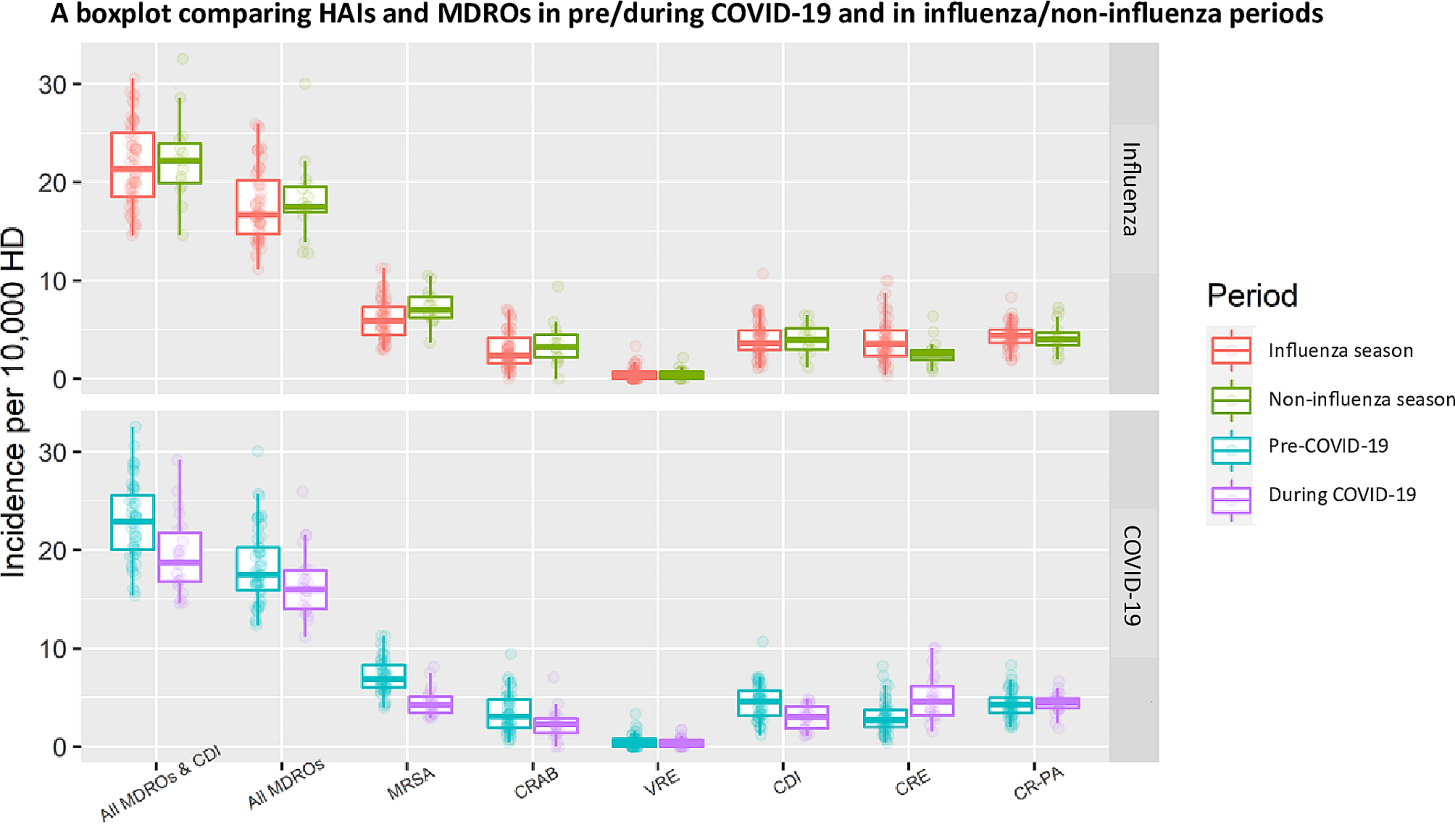
A boxplot comparing HAI and MDROs in pre/during COVID-19 and in influenza and non-influenza periods

A comparable analysis was conducted, examining the infection rates during influenza and non-influenza seasons. The findings revealed no notable differences in the infection rates between these seasons (Table 2). Figure 1 visually illustrates the disparities in the measured parameters in response to both COVID-19 and seasonal influenza.

**Table 2:** The difference in median acquisition rate of multi-drug resistant organisms, hand hygiene compliance, and hospital-acquired bloodstream infection during the influenza and non-influenza seasons between the years 2016–2021

	Influenza seasons (December-February)	Non-influenza seasons (March- November)	Total	*p*-value
Number of hospital days	481,756	1,411,702	1,893,458	
HHC rate [median (Q1-Q3)]	0.7 (0.7–0.8)	0.7 (0.6–0.8)	0.7 (0.6–0.8)	0.386
Non-compliance due to gloves donning [median (Q1-Q3)]	0.5 (0.4–0.5)	0.4 (0.3–0.5)	0.4 (0.3–0.5)	0.357
HHC opportunities [median (Q1-Q3)]	534.5 (267.2–977.0)	658.0 (469.0-1160.0)	654.0 (400.8134.2)	0.151
Overall HHC opportunities	12,234	46,423	58,657	
CRE/10,000 HD [median (Q1-Q3)]	2.5 (2.1–3.1)	3.5 (2.3–4.9)	3.1 (2.3–4.7)	0.065
MRSA/10,000 HD [median (Q1-Q3)]	6.5 (5.6–7.7)	6.2 (4.8–7.5)	6.3 (4.8–7.5)	0.498
CRAB/10,000 HD [median (Q1-Q3)]	2.5 (1.7–3.6)	2.5 (1.7–4.4)	2.5 (1.7–4.3)	0.720
CR-PA/10,000 HD [median (Q1-Q3)]	3.9 (3.5–4.3)	4.4 (3.6-5.0)	4.3 (3.6-5.0)	0.163
VRE/10,000 HD [median (Q1-Q3)]	0.4 (0.0-0.7)	0.4 (0.0-0.8)	0.4 (0.0-0.7)	0.685
CDI/10,000 HD [median (Q1-Q3)]	3.9 (3.1-5.0)	3.9 (2.8–4.9)	3.9 (2.9-5.0)	0.675
All MDROs [median (Q1-Q3)]	17.3 (13.7–18.3)	16.9 (15.6–20.9)	17.1 (14.9–20.0)	0.233
All MDROs & CDI [median (Q1-Q3)]	21.0 (17.4–23.1)	21.9 (18.8–25.4)	21.5 (18.6–24.7)	0.218
HA-BSI/10,000 HD [median (Q1-Q3)]	26.9 (23.2–30.1)	28.0 (24.4–31.2)	27.8 (24.1–31.2)	0.267
CLABSI/1,000 CD [median (Q1-Q3)]	2.3 (1.4-4.0)	3.0 (1.5–4.4)	2.7 (1.5–4.4)	0.563
Overall catheter days	15,869	43,466	59,335	

HHC- hand hygiene compliance, CRE- carbapenem-resistant *Enterobacterales*, MRSA*-* methicillin-resistant *Staphylococcus aureus*, CRAB- carbapenem-resistant *Acinetobacter baumani*, CR-PA-Carbapenem-resistant *Pseudomonas aeruginosa*, VRE- vancomycin resistant *Enterococcus*. CDI- *Clostridioides difficile* infections

## Discussion

The primary finding of the study indicates that the preventive measures implemented during the COVID-19 pandemic led to a reduction in the rate of HAIs and MDROs, which differs from the impact of the influenza season on these types of infections. Specifically, there was a decline in MDRO rates excluding CRE, and in the rate of nosocomial CDI. Notably, this reduction could not be attributed to an improvement in HHC, as HHC remained constant throughout the study period.

Debates persist in the literature concerning the influence of COVID-19 on hospital-acquired infections and the acquisition of MDROs [3–8]. This impact may vary depending on whether patients were diagnosed with COVID-19 upon admission during the pandemic [16]. Despite increased awareness of infection prevention measures and Personal Protective Equipment (PPE) usage, other pandemic-related factors could adversely affect the incidence of such infections such as shortages in healthcare personnel, facility capacity limitations, PPE supply shortages, and increased antibiotic consumption [8, 17, 18]. Additionally, lack of improvement in during the COVID-19 pandemic could be explained by the behavioral component of HCWs in response to the prolonged, exhausting pandemic [9–12]. At times, demonstrating improvement can be challenging when there are already high levels of hygiene measures in place and low rates of HAI and MDROs before the onset of the pandemic.

The reduction in HAIs and MDROs in the current study can be credited to the prompt action to keep and enhance IPAC practices and HHC monitoring at the outset of the pandemic. With slight adjustments, HHC audits were consistently conducted as part of surveillance programs, contributing to the favorable impact on the rate of HAIs.

The lack of improvement in HHC found in this study could be explained by increased workload as opportunities for HHC increase. Glove use, which has been shown to have a negative effect on HHC [19], was not altered by the onset of COVID-19. Previous studies showed varying effects of COVID-19 on HHC, ranging from constant [20] or temporary improvement [21, 22] to worsening [23].

The rise in HA-BSI observed in this study did not coincide with an increase in CLABSI rates. This raises questions about whether the proportional rate of secondary BSI, particularly those stemming from hospital-acquired ventilator-associated pneumonia, was higher during the COVID-19 pandemic. Previous publications have shown an increased rate of VAP/HAP, with some of the studies showing an increase in the overall VAP/HAP rate and others showing an increased rate in COVID-19 patients [24, 25].

The exceptional increase in CRE during COVID-19 can be attributed to the nationwide increase in rates of CRE in Israel since 2016 and is not necessarily due to the COVID-19 pandemic, as this trend was not observed in other MDROs before the pandemic [26]. This increase in CRE could be due to the spread of CRE in the community as demonstrated by comparing these organisms in community and hospital sewage water [27] along with the clonal spread of CRE strains, such as the increase in New Delhi metallo-β-lactamase (NDM) and OXA-48 carbapenemases instead of Klebsiella pneumoniae carbapenemase (KPC)-producing Klebsiella pneumoniae in recent years [28].

The significant decrease in CDI has been reported in previous studies and can be attributed to many factors, some of which were not examined in depth in this study, such as antibiotic use and rigorous environmental cleaning [29, 30]. The lack of improvement in HHC in the current study precludes it from being responsible for the decline in CDI rates.

Remarkably, the decline in MDROs and CDI was not observed during the influenza season. This phenomenon could be attributed to the more substantial impact of the COVID-19 pandemic in comparison to other respiratory viruses. It’s possible that the increased awareness and apprehension surrounding the newly identified SARS-CoV-2 virus played a significant role. Moreover, the ongoing challenges posed by the COVID-19 pandemic, including the emergence of various virus variants with varying transmissibility and severity characteristics when compared to known influenza strains, continued to underscore the importance of vigilance and adherence to infection prevention measures necessary to mitigate transmission risks. These factors collectively contributed to the observed trends in MDROs and CDI rates [20, 31, 32].

Furthermore, we propose the hypothesis that the relatively brief duration of the influenza season, when contrasted with the prolonged period of COVID-19 prevalence, might not have allowed sufficient time for the initiation and consolidation of alterations in HCWs’ behavior and institutional policies. These changes would need time to be translated into measurable differences in the rates of MDROs or HAIs.

This study may have several potential limitations. This is a single-center study that make it difficult to generalize its results to other institutions but conducting it at a large referring hospital may mitigate this limitation. The data in the current study did not distinguish between MDRO, CDI and HA-BSI in COVID-19 patients and COVID-19 free patients, who may have a different likelihood of developing such infections. Also, variables related to patients’ comorbidities were not captured during the study period, but the inclusion of 6 years in the analysis may blur this limitation.

Additionally, data on antimicrobial consumption was not included in the study, as it could potentially influence the rates of MDROs and CDI within the hospital.

## Conclusions

This study indicates that MDRO and CDI rates declined during the COVID-19 pandemic compared to pre-pandemic times, except for increased CRE rates unrelated to the pandemic. This differing impact during the influenza season highlights the different perceptions of these two viruses and the challenge of short-term influenza effects evaluation. The lack of improved HHC suggests that factors like PPE use, enhanced environmental cleaning, and potentially reduced antibiotic consumption may have contributed to lower rates of such infections. However, further detailed studies are needed to explore the specific factors involved in this decline.

## Data Availability

The datasets used and/or analyzed during the current study are available from the corresponding author on reasonable request.

## Abbreviations

COVID-19: coronavirus disease 2019
IPAC: infection prevention and control
HHC: hand hygiene compliance
HAI: hospital acquired infection
MDRO: multidrug-resistant organism
HCW: health care worker
HA-BSI: hospital-acquired bloodstream infection
RHCC: Rambam Health Care Campus
CDI: *Clostridioides difficile* infection
CLABSI: central line associated BSI
HD: hospitals day
CD: catheter day
MRSA: methicillin resistant *Staphylococcus aureus*
CRE: carbapenem-resistant *Enterobacterales*
CRAB: carbapenem resistant *Acinetobacter baumani*
CR-PA: Carbapenem-resistant *Pseudomonas aeruginosa*
VRE: vancomycin resistant *Enterococcus*
